# GSOS-ELM: An RFID-Based Indoor Localization System Using GSO Method and Semi-Supervised Online Sequential ELM

**DOI:** 10.3390/s18071995

**Published:** 2018-06-21

**Authors:** Fagui Liu, Dexiang Zhong

**Affiliations:** School of Computer Science and Engineering, South China University of Technology, Guangzhou 510006, China; fgliu@scut.edu.cn

**Keywords:** radio frequency identification (RFID), indoor localization, Glowworm Swarm Optimization (GSO), semi-supervised online sequential extreme learning machine (SOS-ELM)

## Abstract

With the rapid development of indoor positioning technology, radio frequency identification (RFID) technology has become the preferred solution due to its advantages of non-line-of-sight, non-contact and rapid identification. However, the accuracy of existing RFID indoor positioning algorithms is easily affected by the tag density and algorithm efficiency, and their environmental robustness is not strong enough. In this paper, we have introduced an RFID positioning algorithm based on the Glowworm Swarm Optimization (GSO) fused with semi-supervised online sequential extreme learning machine (SOS-ELM), which is called the GSOS-ELM algorithm. The GSOS-ELM algorithm automatically adjusts the regularization weights of the SOS-ELM algorithm through the GSO algorithm, so that it can quickly obtain the optimal regularization weights under different initial conditions; at the same time, the semi-supervised characteristics of the GSOS-ELM algorithm can significantly reduce the number of labeled reference tags and reduce the cost of positioning systems. In addition, the online learning phase of the GSOS-ELM algorithm can continuously update the system to perceive changes in the environment and resist the environmental interference. We have carried out experiments to study the influence factors and validate the performance, both the simulation and testbed experiment results show that compared with other algorithms, our proposed GSOS-ELM localization system can achieve more accurate positioning results and has certain adaptability to the changes of the environment.

## 1. Introduction

With the development of Internet of Things technology, people’s demand for applications has grown rapidly. Among these technologies, wireless location-aware technologies have shown great activity in both military and civilian applications. Wireless location-aware technologies and services play a more and more important role in people’s daily life. In outdoor location awareness technologies, Global Positioning System (GPS) [1] is the most famous and most representative of location sensing technology and is widely used in military and civilian applications. The demand for indoor location-aware applications is increasing, and there is great potential for indoor real-time and dynamic location-awareness needs. Due to the advantages of non-line-of-sight, non-contact and rapid identification, radio frequency identification (RFID) technology has become the preferred solution to indoor location-aware applications. There are many kinds of location-aware algorithms using RFID, such as Received Signal Strength Indication (RSSI) [2], Angle of Arrival (AOA) [3], Time of Arrival (TOA) [4], Time Division of Arrival (TDOA) [5] and other distance-based RFID positioning methods, but these methods are sensitive to the environment nor their environmental robustness is not strong enough.

At the same time, another kind of RFID positioning method based on scene analysis has attracted a lot of research attention due to its higher adaptability to environment and lower cost. For example, LANDMARC [6] is a classic RFID location-aware algorithm. The algorithm introduces the concept of reference tags to assist the localization. Zhao et al. [7] propose the VIRE algorithm to improve the LANDMARC algorithm by inserting virtual reference tags and introducing the concept of fuzzy maps. Xu et al. [8] use Bayesian probability and *k*-nearest neighbor (KNN) to reduce the location fluctuation and error caused by multipath and environmental interference in LANDMARC. In the scene-based analysis method, the use of neural network methods for RFID positioning has also become another research hotspot. Kung et al. [9] propose a passive RFID indoor positioning scheme that combines LANDMARC scheme with a back propagation (BP) neural network. After LANDMARC location perception, the BP neural network is further used to process location-aware results to obtain more accurate location-aware results. Guo et al. [10] propose an algorithm using radial basis function neural network (RBFNN) for RFID indoor location sensing. The RSSI values and RSSI difference (ΔRSSI) are used as the input of the RBFNN, and the positioning result was obtained. Gholoobi et al. [11] use the weighted *k*-nearest neighbor (WKNN) method to process the captured signal and achieve indoor localization. Mazan et al. [12] design a feed-forward artificial neural network (ANN) to process data and produce estimated coordinates that denote the position of the user. Zou et al. [13] propose an RFID positioning method using an extreme learning machine (ELM), which takes signal strength values as input and coordinates as output. Yang et al [14] propose to use the online extreme learning machine (OS-ELM) to locate the indoor manufacturing execution system. This algorithm uses the known labels in the environment to learn and can adapt to changes in the environment over time, but it requires more tags and higher system costs to reach high positioning accuracy.

In recent years, many studies use evolutionary algorithms to optimize artificial neural networks (ANNs). The optimization of artificial neural networks mainly focuses on three aspects: optimizing the initial weight of the network, optimizing and selecting the network structure and learning algorithms for training. For example, Lin et al. [15] suggest five ANNs in parallel and use the genetic algorithm (GA) [16] to optimize weight values of each network for RFID positioning. Wang et al. [17] proposed a method using particle swarm optimization (PSO) [18] optimized BP neural network for RFID indoor positioning. Kuo et al. [19] proposed an algorithm that uses immune-based feed-forward neural network to learn the relationship between RSSI values and actual locations. Krishnanand et al. [20,21] propose a new type of evolutionary algorithms called Glowworm Swarm Optimization (GSO) algorithm to simulate the behavior of natural fireflies for feeding or courtship. Xu et al. [22] employ the GSO algorithm to optimize the initial weights and biases of the BP neural network. Wang et al. [23] improve the BP neural network using the adaptive-step-size glowworm swarm optimization. Li et al. [24] propose a parallel ensemble learning algorithm based on improved binary glowworm swarm optimization algorithm (IBGSO) and BP neural network. However, for the ELM algorithm, since the weights of the input layer and the biases of the hidden layer are randomly selected and will not be corrected in the training process, in the past ELM optimization research, most studies use different forms of optimization algorithm to optimize the input weights and biases of the ELM to obtain better and more stable models. For example, Zhu et al. [25] use the standard differential evolution algorithm to select and optimize the ELM input weights and hidden layer biases, which is called evolutionary extreme learning machine (E-ELM). Cao et al. [26] use a variety of mutation strategies to improve the standard differential evolution algorithm and proposes an improved version of the E-ELM algorithm, which is called self-adaptive evolutionary extreme learning machine (SaE-ELM). The PSO-ELM fusion algorithm proposed by Xu et al. in literature [27] uses the PSO algorithm to optimize the input weight of the ELM and the bias of the hidden layer. Han et al. [28] have considered the complexity of the ELM model in the optimization, improved the PSO-ELM algorithm, and proposed the ELM algorithm combining with an improved PSO method (IPSO-ELM).

In this paper, we propose an RFID positioning algorithm based on the Glowworm Swarm Optimization (GSO) and semi-supervised online sequential extreme learning machine (SOS-ELM), which is called the GSOS-ELM algorithm. First, we use improved Gaussian filter algorithm to preprocess the RFID data. Then, we use GSO algorithm to optimize the regularization coefficients and train the initial model. Finally, we use the continuously arriving data to update the model and process the user localization requests. The proposed algorithm can quickly obtain the optimal regularization weights under different initial conditions; at the same time, the semi-supervised characteristics can significantly reduce the number of labeled reference tags and reduce the cost. In addition, the online learning phase can continuously update the system to resist the environmental interference. The experimental results show that compared with other algorithms, the proposed localization system can achieve more accurate positioning results and has certain adaptability to the changes of the environment.

The rest of this article is organized as follows: section “Algorithms” introduces the principles of the algorithms; section “Simulation Experiment” explains the algorithm simulation and the simulation results; section “Experimental Evaluation” shows the experimental evaluation results in realistic environment; and section “Conclusion” gives a conclusion of this article and explains our future work.

## 2. Algorithms

In this part, we will introduce the algorithms in detail. First of all, we will present the semi-supervised online sequential extreme learning machine (SOS-ELM); then we will introduce the glowworm swarm optimization (GSO) method; and finally we demonstrate our proposed RFID-based indoor localization system using GSOS-ELM.

### 2.1. Semi-Supervised Online Sequential Extreme Learning Machine

To overcome the problems of traditional BP algorithm such as slow learning rate and local minimum in the process of training single-hidden layer feedforward neural networks (SLFNs), Huang et al. [29,30] proposed a simple SLFNs, which is called Extreme Learning Machine (ELM). Its characteristic is that only the number of hidden layer nodes of a SLFNs needs to be set. Without adjusting the input weight and the bias of the hidden element, the input weights of the SLFNs and the offsets of the hidden layer neurons are randomly given in the ELM. The weight of the output layer can be calculated by the Moore–Penrose generalized inverse of the hidden layer output matrix. The results from ELM algorithm have better generalization performance and its learning speed has been greatly improved compared to traditional neural networks. The basic principle of the ELM algorithm is as follows:

Given a training set with N samples $\left\{\left(x_{i},y_{i}\right)|i=1,2,\cdots ,N\right\}$, where $x_{i}\in R^{n}$ is the input space and $y_{i}\in R^{m}$ is the output space. The output of the ELM model with *L* hidden layer nodes can be expressed as:

(1) $$f\left(x_{i}\right)=h{\left(x_{i}\right)}^{T}\beta =\sum_{i=1}^{L}\beta_{i}G\left(w_{i},b_{i},x_{i}\right)=y_{i},i=1,2,\cdots ,N$$

where $\beta_{i}={\left[\beta_{i1},\beta_{i2},\cdots ,\beta_{in}\right]}^{T}$ is the set of weights between the *i*-th hidden layer neuron and output layer nodes, $w_{i}={\left[w_{i1},w_{i2},\cdots ,w_{im}\right]}^{T}$ is the set of weights connecting the input layer node and the *i*-th hidden layer, $b_{i}$ is the bias term used, G(·) is the activation function.

The above model can be represented as a matrix:

(2) Hβ=Y

where $H={\left(\begin{array}{ccc} G(w_{1},b_{1},x_{1}) & \cdots & G(w_{L},b_{N},x_{1})\\ \vdots & \ddots & \vdots \\ G(w_{1},b_{1},x_{N}) & \cdots & G(w_{L},b_{N},x_{N}) \end{array}\right)}_{N\times L},\beta ={\left[\begin{array}{c} \beta_{1}^{T}\\ \vdots \\ \beta_{L}^{T} \end{array}\right]}_{L\times m},Y={\left[\begin{array}{c} Y_{1}^{T}\\ \vdots \\ Y_{N}^{T} \end{array}\right]}_{N\times m}$.

Weights $w_{i}$ and offsets $b_{i}$ are randomly generated in the model, without training and correction. According to the Moore–Penrose generalized inverse theory [31], solving equations can yield $\beta =H^{+}$, where $H^{+}$ is the Moore–Penrose generalized inverse of matrix *H*.

The most common method of solving the generalized inverse matrix $H^{+}$ is the singular value decomposition method [32]. This method can be used whether $H^{T}H$ is a singular matrix or a nonsingular matrix, and its execution speed is also better than orthogonal iterations method. The solution formula is as follows:

(3) $$\beta =\left\{\begin{array}{c} {\left(H^{T}H\right)}^{-1}H^{T}Y),N>L\\ H^{T}{\left(HH^{T}\right)}^{-1}Y,N<L \end{array}\right.$$

where *N* is the number of rows of matrix *H*, *L* is the number of columns of matrix *H*, that is, the number of hidden layer nodes.

At the same time, to improve the stability of the ELM solution, Huang et al. [33] introduce the structural risk as a regularization term, added the L2 regularization term to the objective function of the ELM, give a ridge regularization version of the ELM algorithm. They convert the original optimization problem to optimization problem:

(4) $$\underset{\beta }{min}\left(\frac{1}{2}{∥H\beta -Y∥}^{2}+\frac{C_{1}}{2}{∥\beta ∥}^{2}\right)$$

where $C_{1}$ is the L2 regularization parameter used to adjust the ratio of structural risk and empirical risk to prevent overfitting. Solving Equation (4) results in Equation (5):

(5) $$\beta^{*}=\left\{\begin{array}{c} {\left(H^{T}H+\frac{I_{L\times L}}{C_{1}}\right)}^{-1}H^{T}Y,N>L\\ H^{T}{\left(HH^{T}+\frac{I_{N\times N}}{C_{1}}\right)}^{-1}Y,N<L \end{array}\right.$$

where *N* is the number of rows of matrix *H*, *L* is the number of columns of matrix *H*, and $I_{L\times L}$ and $I_{N\times N}$ are identity matrices.

To solve the problems of excessive training capacity and long training time when training data is large, Liang et al. [34] improved the batch learning ELM algorithm and proposed an online sequential learning ELM algorithm (OS-ELM). The incremental approach learns new knowledge from data that arrives one by one or from chunk by chunk, and after the current data training is completed, the original data can be discarded. Furthermore, to solve the problem of labeled samples shortage, Huang et al. [35] propose a semi-supervised extreme learning machine (SS-ELM) for semi-supervised learning, which reduces the number of labeled samples and uses unlabeled samples to avoid the high cost of sample labels.

Jia et al. [36] comprehensively consider the merits of empirical risk and structural risk in SS-ELM algorithm and the advantage of incremental learning in OS-ELM algorithm, proposed a semi-supervised online sequential extreme learning machine (SOS-ELM), which maintains generalization ability to take online learning at the same time. The description of the SOS-ELM algorithm is as follows:

Given a data set containing the labeled samples $\left\{\left(x_{i},y_{i}\right)|x_{i}\in R^{n},y_{i}\in R^{m},i=1,2,\cdots ,N_{l}\right\}$ and the unlabeled sample $\left\{x_{i}^{\prime }|x_{i}^{\prime }\in R^{n},i=1,2,\cdots ,N_{u}\right\}$, the number of hidden neurons is *L*, the activation function is g(x), the hyperparameters $C_{1},C_{2}$ correspond to the L2 regularization term and the regularization term of the manifold respectively, *J* is a diagonal matrix, $\left[J_{ii}\right]=E_{i},i=1,2,\cdots ,l$, the remaining elements are 0, $E_{i}$ is the penalty factor, and is set to 1 when used to solve the regression problem. The specific steps of the SOS-ELM algorithm are described in Procedure 1.
**Procedure 1** Semi-Supervised Online Sequential Extreme Learning Machine**Initialization phase:** Let the training initial data set size be $N_{0}$, then the initialized data set is $\left\{\left(x_{i},y_{i}\right)\;or\;x_{i}^{\prime }|i=1,2,\cdots ,N_{0}\right\}$.
**Step 1.1:** Randomly generate the hidden layer weights $w_{i}$ and offsets $b_{i}$.**Step 1.2:** Record the labeled sample $n_{l}$ and unlabeled sample $n_{u}$, calculate the similarity matrix of the sample:
(6)$$W={\left[w_{ij}\right]}_{u\times u}$$
where $w_{ij}$ is the similarity between sample *i* and sample *j*, $w_{ij}$ = $w_{ji}$.**Step 1.3:** Calculate the initial diagonal matrix $J_{0}$ and the Laplacian matrix $L_{{ℵ}_{0}}$:
(7)$$L_{{ℵ}_{0}}=D_{{ℵ}_{0}}-W_{{ℵ}_{0}}$$
where $D_{{ℵ}_{0}}$ is a diagonal matrix, the *i*-th diagonal element is $\sum_{j=1}^{u}w_{ij}$.**Step 1.4:** Calculate the initial output weight:
(8)$$\beta^{0}=P_{0}H_{0}^{T}J_{0}Y_{0}$$
where $P_{0}={\left(C_{1}I+H_{0}^{T}J_{0}H_{0}+C_{2}H_{0}^{T}L_{{ℵ}_{0}}H_{0}\right)}^{-1}$**Step 1.5:** Let k=0.**Online learning phase:** The (k+1)-th new data block arrives:
**Step 2.1:** Record the labeled samples $n_{l}$ and unlabeled samples $n_{u}$, calculate the diagonal matrix $J_{k+1}$ and Laplacian matrix $L_{{ℵ}_{k+1}}$.**Step 2.2:** Calculate $P_{k+1}$ and output weight $\beta^{k+1}$:
(9)$$\begin{array}{cc} P_{k+1} & =P_{k}-P_{k}H_{k+1}^{T}{\left(C_{1}I+\left(J_{k+1}+C_{2}L_{{ℵ}_{k+1}}\right)H_{k+1}^{T}P_{k}H_{k+1}^{T}\right)}^{-1}\left(J_{k+1}+C_{2}L_{{ℵ}_{k+1}}\right)H_{k+1}P_{k} \end{array}$$
(10)$$\begin{array}{cc} \beta^{k+1} & =\beta^{k}+P_{k+1}H_{k+1}^{T}\left(J_{k+1}Y_{k+1}-\left(J_{k+1}+C_{2}L_{{ℵ}_{k+1}}\right)H_{k+1}\beta^{k}\right) \end{array}$$**Step 2.3:** Let k=k+1, return to **Step 2.1**.

### 2.2. Glowworm Swarm Optimization Method

The Glowworm Swarm Optimization (GSO) algorithm simulates the glow behavior of the firefly in nature, uses its luminescence properties to find partners based on its search area, and moves to a firefly with a superior position in the neighborhood structure to achieve evolution.

The relative brightness of the firefly is $I=I_{0}\times e^{-\gamma r_{ij}}$, where $I_{0}$ is the maximum fluorescent intensity of the firefly, i.e., the fluorescence intensity of itself (r=0), which is related to the objective function value; γ is the light intensity absorption coefficient, to reflect the fact that fluorescence gradually decreases with distance increases and absorption of the media; $r_{ij}$ is the spatial distance between fireflies *i* and *j*.

The degree of attraction of fireflies is $\beta =\beta_{0}\times e^{-\gamma r_{ij}^{2}}$, where $\beta_{0}$ is the maximum degree of attraction, that is, the degree of attraction at the light source (r=0).

The position of the firefly i being attracted to the firefly j is updated by $x_{i}=x_{i}+\beta \times \left(x_{j}-x_{i}\right)+\alpha \times \left(rand-1/2\right)$, where $x_{i}$, $x_{j}$ are the spatial positions of the fireflies *i* and *j*; α is the constant step length factor on [0, 1]; rand is a random factor that follows uniform distribution on [0, 1].

The specific steps of the GSO algorithm are described as Procedure 2.
**Procedure 2** Glowworm Swarm Optimization Method**Step 1:** Initialize the basic parameters: set the number of fireflies *m*, the maximum attraction $\beta_{0}$, the light absorption coefficient γ, the step length factor α, the maximum number of iterations maxT or the search accuracy ϵ.**Step 2:** Randomly initialize the position of the firefly and calculate the target value of the firefly as its maximum fluorescent intensity $I_{0}$.**Step 3:** Calculate the relative brightness *I* and the attractiveness β of the firefly in the population and determine the direction of movement of the firefly based on the relative brightness:
(11)$$\begin{array}{cc} I & =I_{0}\times e^{-\gamma r_{ij}} \end{array}$$
(12)$$\begin{array}{cc} \beta & =\beta_{0}\times e^{-\gamma r_{ij}^{2}} \end{array}$$
where γ is the light intensity absorption coefficient and $r_{ij}$ is the spatial distance between fireflies *i* and *j*.**Step 4:** Update the spatial position of the firefly:
(13)$$x_{i}=x_{i}+\beta \times \left(x_{j}-x_{i}\right)+\alpha \times \left(rand-1/2\right)$$
where $x_{i}$, $x_{j}$ are the spatial positions of the fireflies *i* and *j*; α is the constant step length factor on [0, 1]; rand is a random factor that follows uniform distribution on [0, 1].**Step 5:** Randomly disturb the firefly at the best position.**Step 6:** Recalculate the firefly’s brightness $I_{new}$ based on the location of the updated firefly.**Step 7:** When the search accuracy is satisfied, or the maximum number of searches is reached, skip to **Step 8**; otherwise, increase the number of searches by 1 and skip to **Step 3** for the next search.**Step 8:** Output global extreme points and optimal individual values.

### 2.3. RFID-Based Indoor Localization System

Based on the semi-supervised online sequential extreme learning machine (SOS-ELM) and Glowworm Swarm Optimization (GSO) method, this paper proposes an RFID indoor localization algorithm that combines GSO algorithm and SOS-ELM algorithm, which is called the GSOS-ELM algorithm.

**Figure 1:**
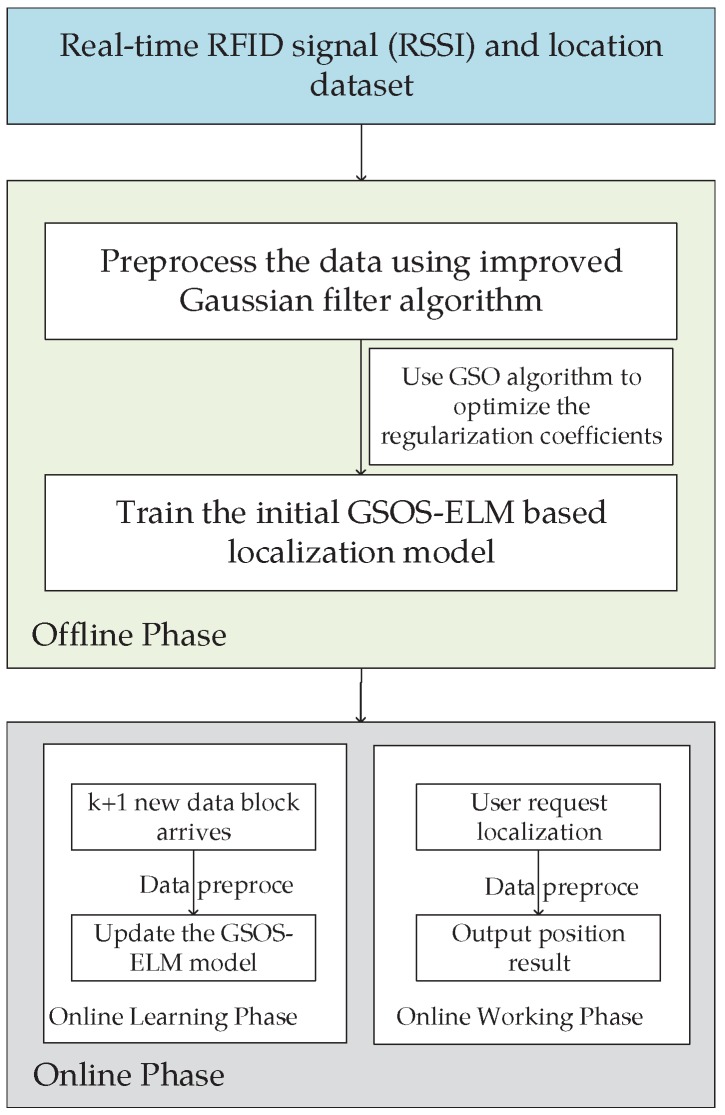
The Framework of RFID Localization System Using GSOS-ELM.

The framework of the proposed system is shown in Figure 1. The system includes *n* readers, *l* reference tag with known position, *u* reference tags with unknown positions, the signal strength of the *i*-th reader reading the tag is $RSSI_{i}$, the first *l* elements of diagonal matrix *J* are set to 1 and the following *u* elements are 0.

The specific steps of the GSOS-ELM system are described as Procedure 3, it is divided into the offline phase and the online phase.
**Procedure 3** Semi-Supervised Online Sequential Extreme Learning Machine**Offline phase:****Step 1.1:** Preprocessing the data using an improved Gaussian filter algorithm: (i): The *i*-th reader repeatedly reads the signal strength of the same tag for a total of *N* times, and records the signal strength of the *k*-th read signal as $RSSI_{ik},k=1,2,\cdots ,N$; (ii): Calculate the variance $\delta^{2}$ of the RSSI value:
(14)$$\delta^{2}=\frac{1}{N-1}\sum_{k=1}^{N}{\left(RSSI_{ik}-\bar{A}\right)}^{2}$$
where $\bar{A}=\frac{1}{N}\sum_{k=1}^{N}RSSI_{ik}$ (iii): For the k-th signal strength $RSSI_{ik}$, perform culling if $|RSSI_{ik}-\bar{A}|>3\delta$, finally an RSSI set of size *m* is obtained, and the average value of the RSSI set is calculated as the average signal strength:
(15)$$\bar{RSSI_{l}}=\frac{1}{m}\sum_{k=1}^{m}RSSI_{ik}$$**Step 1.2:** Determine the number of hidden neurons of the GSOS-ELM algorithm, the activation function g(x), the regularization coefficients $C_{1}$ and $C_{2}$, and generate hidden layer weights $w_{i}$ and offsets $b_{i}$;**Step 1.3:** The initial data set size is $N_{0}$, record the labeled sample $n_{l}$ and the unlabeled sample $n_{u}$, calculate the similarity matrix $W_{{ℵ}_{0}}$ of the sample:
(16)$$W_{{ℵ}_{0}}={\left[w_{ij}\right]}_{u\times u}$$
where $w_{ij}$ is the similarity between sample *i* and sample *j*, the measure formula is:
(17)$$w_{ij}=e^{-{∥x_{i}-x_{j}∥}^{2}/\left(2\delta^{2}\right)}$$**Step 1.4:** Calculate the initial Laplacian matrix $L_{{ℵ}_{0}}$:
(18)$$L_{{ℵ}_{0}}=D_{{ℵ}_{0}}-W_{{ℵ}_{0}}$$
where $D_{{ℵ}_{0}}$ is a diagonal matrix, the *i*-th diagonal element is $\sum_{j=1}^{u}w_{ij}$.**Step 1.5:** Calculate the initial output weight $\beta^{0_{t}}=P_{0}H_{0}^{T}J_{0}Y_{0}$, where $P_{0}=\left(C_{1}I+H_{0}^{T}J_{0}H_{0}+C_{2}H_{0}^{T}L_{{ℵ}_{0}}H_{0}\right)$, $J_{0}$ is a diagonal matrix, $[J_{ii}]=1,i=1,2,\cdots ,l$, the remaining elements are 0, get the output matrix $\hat{Y}$ of the labeled sample and the real value *Y*;**Step 1.6:** Optimize the regularization coefficients $C_{1}$ and $C_{2}$ according to the specific steps in Procedure 2, the fitness function is:
(19)$$fitness=\frac{1}{\sum_{i=1}^{l}∥\hat{Y_{l}}-Y_{i}∥}$$**Step 1.7:** The optimized initial output weight $\beta^{0}$ is output to online learning phase and online working phase.**Online phase:** The online phase includes online learning phase and online working phase, they can be performed parallel.
**Online learning phase:** The k+1 new data block arrives:**Step 2.1.1:** Record the labeled samples $n_{l}$ and unlabeled samples $n_{u}$, use improved Gaussian filter to process the data and calculate the Laplacian matrix $L_{{ℵ}_{k+1}}$.**Step 2.1.2:** Calculate $P_{k+1}$ and output weight $\beta^{k+1}$:
(20)$$\begin{array}{cc} P_{k+1} & =P_{k}-P_{k}H_{k+1}^{T}{\left(C_{1}I+\left(J_{k+1}+C_{2}L_{{ℵ}_{k+1}}\right)H_{k+1}^{T}P_{k}H_{k+1}^{T}\right)}^{-1}\left(J_{k+1}+C_{2}L_{{ℵ}_{k+1}}\right)H_{k+1}P_{k} \end{array}$$
(21)$$\begin{array}{cc} \beta^{k+1} & =\beta^{k}+P_{k+1}H_{k+1}^{T}\left(J_{k+1}Y_{k+1}-\left(J_{k+1}+C_{2}L_{{ℵ}_{k+1}}\right)H_{k+1}\beta^{k}\right) \end{array}$$**Step 2.1.3:** Let k=k+1, return to **Step 2.1.1**.**Online working phase:****Step 2.2.1:** The user requests positioning from the server through the client and sends the signal strength RSSI information at the unknown location to the server.**Step 2.2.2:** The server uses the RSSI information sent from the client as input to the current GSOS-ELM model to estimate the positioning result and send it back to the client.

## 3. Simulation Experiment

**Figure 2:**
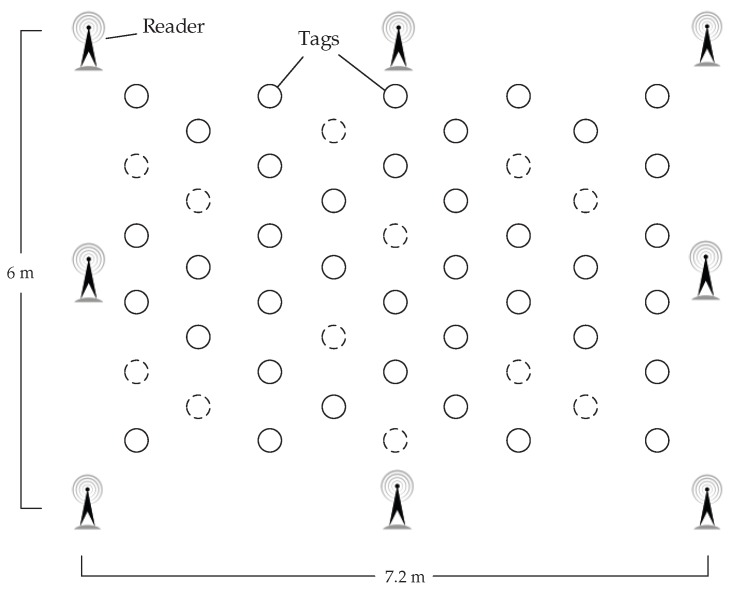
Simulation Experiment Layout.

As shown in Figure 2, we have performed a simulation experiment on the MATLAB platform and simulated a 6 m × 7.2 m area, the reference readers are placed in the corners and the edges, and the RFID tags are distributed in the region. The objective of our simulation experiment is to analyze the impact factors and compare the localization effect in different environments. The experimental configurations are as follows: (1) operating systems: Windows 10 x64 v1803; (2) CPU: Intel(R) Core(TM) i3-4160 @ 3.60 GHz; (3) memory: 4 GB; and (4) software: MATLAB R2015b.

In the simulation experiment, we use the log-normal path loss model to model the attenuation of the signal in the indoor environment with distance:

(22) $$PL\left(d\right)=PL\left(d_{0}\right)+10n\log_{10}\frac{d}{d_{0}}+X_{\delta }$$

where $d_{0}$ is the reference distance, *n* is the path loss factor, *d* is the distance between the sender and the receiver of the signal, and $X_{\delta }$ is zero mean Gaussian random variable with standard deviation of δ.

The RSSI value of RFID can be expressed by Equation (23):

(23) $$RSSI\left(d\right)=P_{t}+G_{t}-PL\left(d\right)$$

where $P_{t}$ is the transmit power, and $G_{t}$ denotes the antenna gain of transmit node.

Since the $G_{t}$ is fixed, combine Equations (22) and (23), we can get Equation (24):

(24) $$RSSI\left(d\right)=RSSI\left(d_{0}\right)-10n\log_{10}\frac{d}{d_{0}}+X_{\delta }$$

where $d_{0}$ is the reference distance and *n* is the path loss factor. According to the analysis in [37,38], we set $RSSI(d_{0})=-45$ dBm, n=2,δ=2 in the simulation experiment.

The experimental error is defined as the Euclidean distance between the estimated target tag position $\left(x_{e},y_{e}\right)$ and the actual target tag position $\left(x_{t},y_{t}\right)$, the *i*-th error result is denoted as $\rho_{i}$:

(25) $$\rho_{i}=\sqrt{\left({\left(x_{e}-x_{t}\right)}^{2}+{\left(y_{e}-y_{t}\right)}^{2}\right)}$$

The average error of the system is $\bar{\rho_{i}}=\sum_{i=1}^{n}\rho_{i}/n$, where *n* is the number of target tags in the positioning process.

We set the number of hidden layer nodes of the GSOS-ELM model to L=100 and the activation function g(x) to sigmoid. We use the *k*-fold cross-validation to evaluate the performances. The samples are randomly split into *k* subsets equally; then, the subsets are divided into two sets, the testing set with only one subset and the training set with the reset (k−1) subsets; and here we set k=10. After the division of training set and testing set, we further divide the samples into two groups, one group with labels, the rest are unlabeled.

### 3.1. Influence Factors

The main factors influencing the positioning result include the density of reference tags (Δ), the number of reference readers (*N*), the proportion of labeled samples (%), the data preprocessing, and the placement of the reference tags. In the following sections, we will analyze the impact of each factor respectively.

#### 3.1.1. Density of Reference Tags

In this experiment, we set the number of readers to N=8 and the proportion of labeled samples to 40%. We divide the reference tag density into 1.0 m, 0.8 m, 0.5 m and 0.3 m.

**Figure 3:**
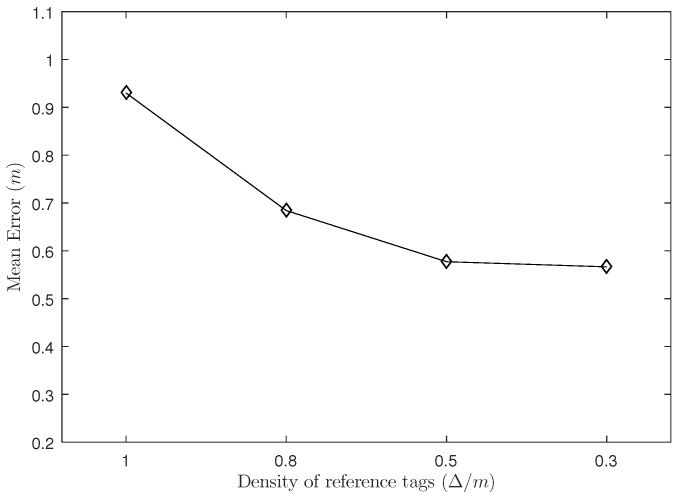
The Influence of Density of Reference Tags.

As shown in Figure 3, when the reference tag density changes from 1.0 m to 0.8 m and 0.5 m, the positioning error decreases significantly, but when the density of reference tags is changed from 0.5 m to 0.3 m, the positioning error does not change much. Because the number of reference tags in the unit area increases as the density of the reference tag increases, thereby reducing the positioning error and obtaining a more accurate positioning result; but when the reference tags reach to a relative high density (here is 0.3 m), it may contain redundant information compared to relatively low density situation (0.5 m) and do not increase the accuracy significantly.

#### 3.1.2. The Number of Readers

In this experiment, we set the density of reference tags to Δ=0.5 m and the proportion of labeled samples to 40%. As shown in Figure 2, we place reference readers in the corners and the edges of the system; the number of reference readers is 4, 6, 8 and 12 respectively.

**Figure 4:**
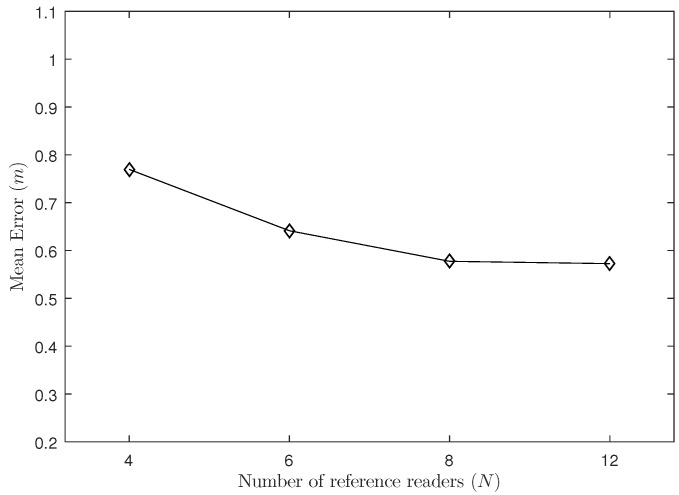
The Influence of the Number of Readers.

As shown in Figure 4, the positioning error of the system decreases as the number of reference readers increases. When the number of reader increases from 4 to 6 and from 6 to 8, the localization accuracy improves noticeably, but when the number of reference readers increases to a certain degree and provides duplicate data, it does not change much on the positioning error, and the increasing in the number of readers will obviously increase the cost of the entire positioning system.

#### 3.1.3. The Proportion of Labeled Samples

In this experiment, we analyze the influence of the labeled samples proportion to the GSOS-ELM algorithm. We set the number of readers to N=8 and the density of reference tags to Δ=0.5 m. The proportion of labeled samples is 20%, 40%, 60%, and 80%, respectively.

**Figure 5:**
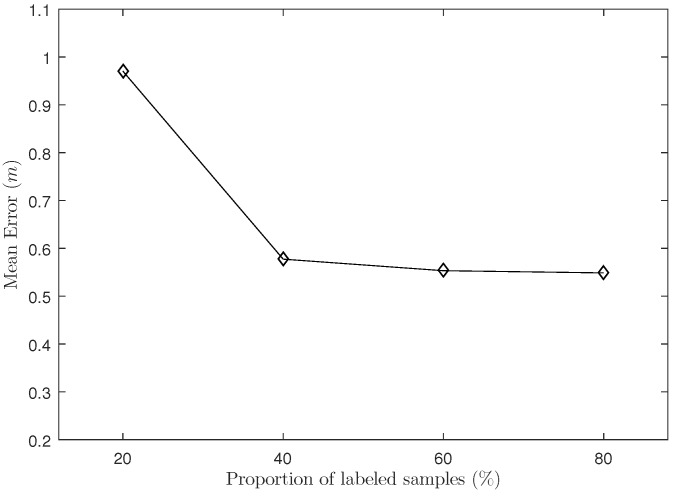
The Influence of Proportion of Labeled Samples.

As shown in Figure 5, the performance of the positioning system increases most significantly when the proportion of the labeled samples increases from 20% to 40%, but the positioning error does not change much as 40% is increased to 60% and 80%. Because when the labeled samples at a low proportion, the increasing of labeled information will significantly improve the non-regularization term of PSOS-ELM algorithm and reduce positioning error; but when the proportion of labeled samples reaches a high degree, the more labeled samples and less unlabeled samples may balance the non-regularization term and regularization term, and the localization accuracy does not change much.

#### 3.1.4. Preprocessing and Reference Tags Placement

In this experiment, we study the influence of the improved Gaussian preprocessing and the reference tags placement method on the positioning effect of the algorithm. We set the number of readers to N=8, the density of reference tags to Δ=0.5 m and the proportion of labeled samples to 40%. The reference tags placement method changes without changing the total number of reference tags, which are squares, rectangles, and equilateral triangles.

As shown in Table 1, the performance of the GSOS-ELM algorithm in positioning has been improved by the pre-processing process. The average error has been improved from 0.6439 m to 0.5774 m, and the standard deviation has decreased from 0.7395 m to 0.6496 m. As shown in Table 2, the placement of the reference tag also has a certain impact on the GSOS-ELM algorithm, when the tag is placed in a rectangular manner, the performance of the algorithm is reduced significantly compared to square and equilateral triangle manner. At the same time, the equilateral triangle placement method has a certain improvement to the square placement method, and the average error decreases from 0.6568 m to 0.5774 m.

### 3.2. Comparison with Other Methods

According to the results from Section 3.1, we set the default condition to: the density of the reference tag is Δ=0.5 m, the number of readers is N=8, the proportion of labeled samples is 40% and the placement of the reference tags is equilateral triangle.

In this section, we compare the proposed GSOS-ELM algorithm with the NN-Based algorithm proposed in [9], the FA-OSELM algorithm proposed in [39], and the NMDS algorithm proposed in [40]. The NN-Based algorithm uses a BP neural network to enhance the LANDMARC algorithm [6]; the FA-OSELM algorithm uses incremental data to update the original model to a transferred model; and the NMDS algorithm combines nonmetric multidimensional scaling algorithm and fingerprinting algorithm to archive localization. We implement these algorithms and perform experiments in the same simulated space. The parameters setting for these methods is described in Table 3.

The comparison results are shown in Table 4. The proposed GSOS-ELM algorithm has certain advantages over other algorithms in terms of average error and stability. Compared with the NN-Based algorithm, FA-OSELM algorithm, and NMDS algorithm, the average error of our proposed GSOS-ELM algorithm improves by 13.46%, 16.56% and 11.94%, respectively. Also, we can learn that the max error is higher at 1.8447 m, this is because when the target tag falls into the edges and corners, the decrease in the reference tags leads to the increase in the error. For the average execution time of algorithms, as shown in Table 5, both GSOS-ELM and FA-OSELM reach a better efficiency than NN-Based method and NMDS method. GSOS-ELM takes 17.6613 s and FA-OSELM requires 15.0384 s in average execution time, hence GSOS-ELM is slightly slower than FA-OSELM. GSOS-ELM reduces the average execution time to 21.03% of NN-Based and 40.41% of NMDS.

**Figure 6:**
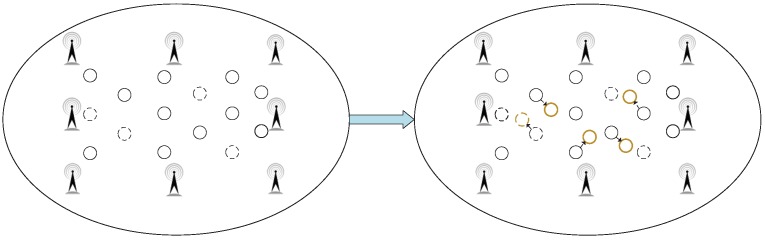
The Dynamic Changes to the Simulation Environment.

**Figure 7:**
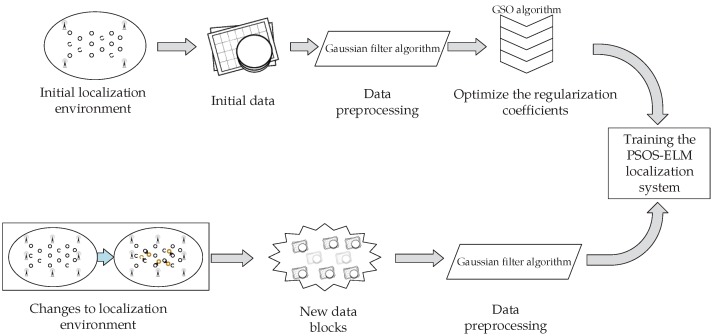
The Steps to Process the Dynamic Changes.

At the same time, as shown in Figure 6, to verify the adaptability of the GSOS-ELM positioning algorithm in the environment, we move some labeled reference tags during the experiment to observe the average error of the positioning system. The specific steps to process the dynamic changes are shown in Figure 7, when the localization environment changes and the new data blocks arrive, we use improved Gaussian filter algorithm mentioned in Procedure 3 to preprocess the data and update the initial PSOS-ELM.

As shown in Table 6, after moving the reference tags, the average error of the GSOS-ELM algorithm increases from 0.5774 m to 0.6428 m, and the increasing rate is 11.33%. At the same time, the average error increasing rate of the FA-OSELM algorithm using the online learning method is 12.69%. However, the average error of the NN-Based and NMDS algorithms without online learning phase raise significantly, with the increasing rates of 28.25% and 25.55%, respectively.

## 4. Experimental Evaluation

### 4.1. Experiment Setting

To further demonstrate the appropriateness of the proposed method under static and dynamics environments compared to the other methods, we have conducted several realistic experiments in Guangzhou Research Institute of O-M-E Technology.

**Figure 8:**
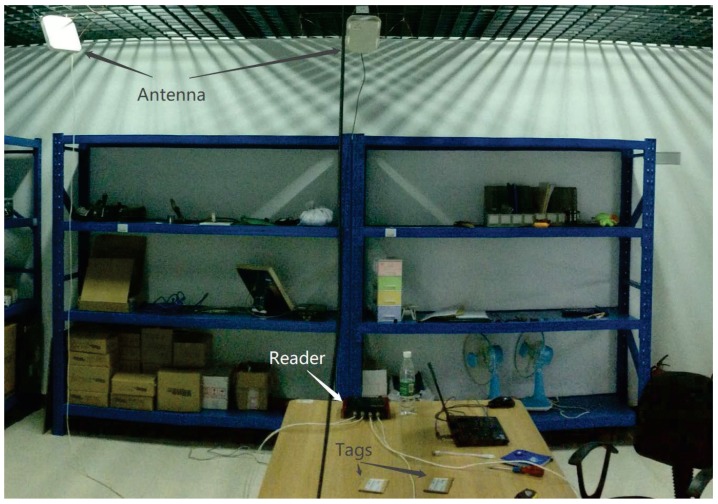
Experimental Setup in Realistic Environment.

**Figure 9:**
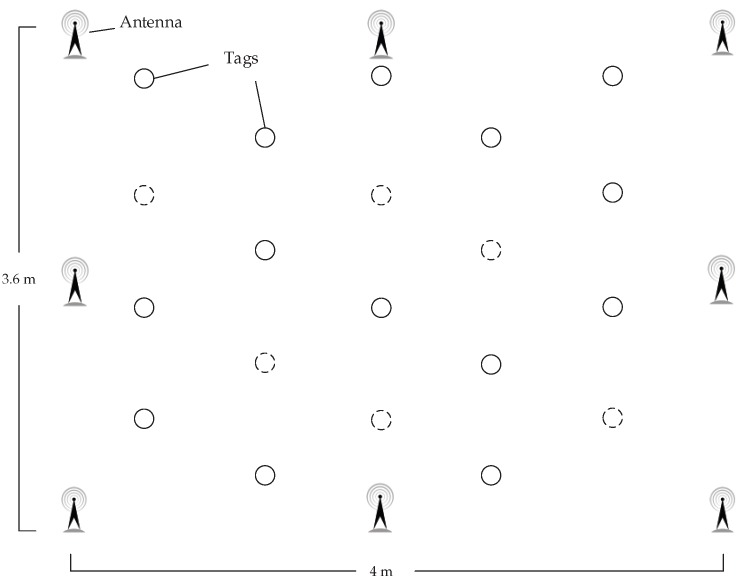
Realistic Experiment Layout.

The reader model we used is Alien ALR-9900+ [41], the main working frequency is 920 MHz, the maximum power strength is 30.7 dBm. We use two kinds of antennas, their model are Alien ALR-8696-C [42] (8.5 dBic gain) and ALR-9611-CR [43] (6 dBic gain). Our experiment setting is shown in Figure 8 and Figure 9, we finish the experiments under a realistic environment with 4.00 m long and 3.63 m wide, we place 8 antennas in the corners and edges and 20 passive RFID tags inside the area. The computer communicates with the ALR-9900+ reader via TCP/IP protocol. Same as previous, the parameters setting is in Table 3, the proportion of labeled samples is 40%, and the 10-fold cross-validation is adopted to evaluate the performances.

### 4.2. Experiment Results

As shown in Table 7, the proposed GSOS-ELM algorithm has certain improvement in average error and standard deviation. The average error of our proposed GSOS-ELM method overcomes by a rate of 15.18%, 18.07% and 12.45% over NN-Based method, FA-OSELM method and NMDS method, respectively. We can also find that the maximum localization error is significantly higher at 1.2489 m, it is due to the reduced reference tags when target tag is in the edges and corners, leading to the higher localization error. The comparison of average execution time is shown in Table 8, we can learn that GSOS-ELM and FA-OSELM take less average execution time than NN-Based method and NMDS method, while GSOS-ELM requires 9.7748 s and FA-OSELM is 1.3017 s faster. The average execution time of GSOS-ELM method is 5.73 times less than NN-Based method and 2.58 times less than NMDS method.

**Figure 10:**
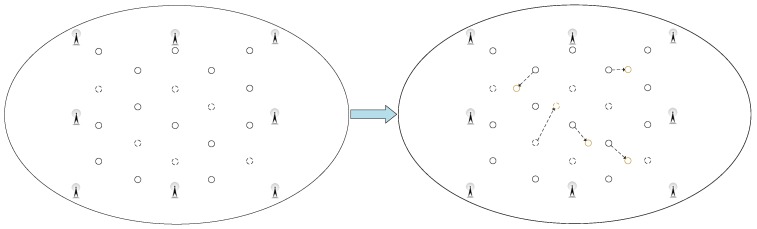
The Dynamic Changes to the Realistic Environment.

At the same time, as shown in Figure 10, we move some labeled reference tags to checkout the adaptability of the algorithms in realistic environment.

The comparison results are in Table 9, after moving the reference tag, the average error of proposed GSOS-ELM algorithm raises from 0.4302 m up to 0.4851 m, with an increasing rate of 12.76%. Meanwhile, the average error raising rate of the FA-OSELM algorithm with the online learning phase is 13.54%, with not much differences. However, the average error of the NN-Based and NMDS algorithms raise significantly, with the rates of 30.78% and 28.14%, respectively. The experimental results show that adopting online learning method can improve the adaptability of the algorithm in the environment.

## 5. Conclusions

This paper proposes an RFID positioning algorithm, which is called the GSOS-ELM algorithm. It is a fusion semi-supervised online sequential extreme learning machine (SOS-ELM) based on the Glowworm Swarm Optimization (GSO), aiming at improving the disadvantages of the existing RFID indoor positioning algorithms, which are susceptible to the tag density and algorithm efficiency, and lack of environmental adaptability. The GSOS-ELM algorithm uses the semi-supervised method to reduce the number of labeled reference tags and the cost of positioning systems; and we use the GSO method to adjusts the regularization weights of the SOS-ELM algorithm, so that it can quickly obtain the optimal regularization weights under different initial conditions. In addition, the online learning phase of the GSOS-ELM algorithm can continuously update the system to perceive changes in the environment and resist the environmental interference.

In the simulation section, we have studied the influence factors of the GSOS-ELM algorithm, including the reference tag density, the number of reference readers, the proportion of labeled samples, data preprocessing and the placement of reference tags. The results show that increasing the proportion of labeled samples, the density of readers and reference tags can increase the localization accuracy, but the rate of increase gradually decreases and the cost of the system raises. Besides, we have given simulation experiment to compare GSOS-ELM method with the other methods and carried out testbed experiment to evaluate our proposed method. Both the simulation and the testbed experiment results have shown that, compared with other algorithms, our proposed algorithm has certain advantages in average error; and the smaller increase in localization errors shows that it has certain adaptability to the changes of the environment.

However, the proposed method does not solve the problem that the localization error is higher when the target tag falls into the edges and corners. Our further work will focus on solving this problem and analyzing how the environment and the location of the antennas affect the level of electromagnetic radiation.

## Figures and Tables

**Table 1:** The Influence of Preprocessing.

Preprocessing	Min Error (m)	Max Error (m)	Average Error (m)	Standard Deviation (m)
Without preprocessing	0.1076	2.1157	0.6439	0.7395
With preprocessing	0.0973	1.8447	0.5774	0.6496

**Table 2:** The Influence of Label Placement.

Reference Tags Placement	Min Error (m)	Max Error (m)	Average Error (m)	Standard Deviation (m)
Square	0.1064	2.2497	0.6568	0.6832
Rectangle	0.1634	2.7769	0.9795	0.8645
Equilateral triangle	0.0973	1.8447	0.5774	0.6496

**Table 3:** Parameters Setting for Proposed Method and Compared Methods.

Method	Parameters Setting
GSOS-ELM	Activation function: sigmoid, L=100
NN-Based	Activation function: sigmoid, L=100, number of neighbors k=4
FA-OSELM	Activation function: RBF, L=350, regularization factor $C=2^{-6}$
NMDS	Goodness fit threshold $\epsilon =10^{-4}$, maximum number of iterations $k_{max}=200$

**Table 4:** Comparison Result under Simulation Environment.

Method	Min Error (m)	Max Error (m)	Average Error (m)	Standard Deviation (m)
GSOS-ELM	0.0973	1.8447	0.5774	0.6496
NN-Based	0.1103	2.4829	0.6672	0.8574
FA-OSELM	0.0935	2.3341	0.6920	0.7933
NMDS	0.1104	2.0745	0.6557	0.7202

**Table 5:** Comparison of Average Execution Time under Simulation Environment.

Method	GSOS-ELM	NN-Based	FA-OSELM	NMDS
**Average Execution Time (s)**	17.6613	83.9932	15.0384	37.2103

**Table 6:** Comparison Result under Dynamic Simulation Environment.

Method	Min Error (m)	Max Error (m)	Average Error (m)	Standard Deviation (m)
GSOS-ELM	0.0997	2.0408	0.6428	0.7155
NN-Based	0.1246	2.6368	0.8557	0.9149
FA-OSELM	0.1041	2.4076	0.7798	0.8581
NMDS	0.1227	2.3718	0.8232	0.8061

**Table 7:** Comparison Results under Realistic Environment.

Method	Min Error (m)	Max Error (m)	Average Error (m)	Standard Deviation (m)
GSOS-ELM	0.0872	1.2489	0.4302	0.4837
NN-Based	0.0974	1.6307	0.5072	0.5704
FA-OSELM	0.0892	1.5513	0.5251	0.5481
NMDS	0.0992	1.4877	0.4914	0.5113

**Table 8:** Comparison of Average Execution Time under Realistic Environment.

Method	GSOS-ELM	NN-Based	FA-OSELM	NMDS
**Average Execution Time (s)**	9.7748	56.0564	8.4731	21.8764

**Table 9:** Comparison Result under Dynamic Realistic Environment.

Method	Min Error (m)	Max Error (m)	Average Error (m)	Standard Deviation (m)
GSOS-ELM	0.0882	1.3258	0.4851	0.5204
NN-Based	0.0993	1.7434	0.6633	0.6620
FA-OSELM	0.0931	1.6041	0.5962	0.5685
NMDS	0.1064	1.5381	0.6297	0.5828
